# A Case Report of Anorexia Nervosa - the “perfect“ woman

**DOI:** 10.1192/j.eurpsy.2022.1495

**Published:** 2022-09-01

**Authors:** M. Viseu, A. Oliveira, M. Barbosa Pinto, R. Sousa

**Affiliations:** 1 University Hospital Center of Algarve, Portugal, Department Of Psychiatry And Mental Health – Faro, Faro, Portugal; 2 USF Ria Formosa, Ars Algarve, Faro, Portugal

**Keywords:** Anorexia nervosa, eating behavior disorder

## Abstract

**Introduction:**

Anorexia nervosa (AN) is an eating behavior disorder characterized by intense fear of gaining weight or persistent behavior that interferes with weight gain, with caloric intake restriction and secondary loss of body weight. It can affect up to 4% of women during their lifetime and is responsible for one of the highest mortality rates from psychiatric disorders.

**Objectives:**

Review of the literature and exposure of a case report of AN in a woman with high level of stress at work.

**Methods:**

Case report and nonsystematic review using databases such as PubMed and UpToDate.

**Results:**

Caucasian woman, 31-year-old, PhD in biology, who works in a multinational company. No personal or family history of psychiatric disorder. She was observed in the psychiatry emergency department, due to low weight, caloric restriction and intense physical exercise, maladaptive personality traits related to perfectionism and control were found. She began follow-up with a multidisciplinary team, but there was a need for hospitalization due to clinical deterioration with BMI of 11. After 6 months, she continued to follow up at the consultations and, despite refusing psychotropic drugs, she maintains psychotherapy and presents clinical improvement (BMI - 17).

**Conclusions:**

Eating behavior disorders are chronic and difficult to treat diseases that are more frequent among people subject to high levels of stress. This case represents a restrictive AN in a woman with multiple risk factors: athlete, perfectionist, with stressful work and life events and restricted interpersonal and affective relationships.

**Disclosure:**

No significant relationships.

